# The Global Burden of Mental, Neurological and Substance Use Disorders: An Analysis from the Global Burden of Disease Study 2010

**DOI:** 10.1371/journal.pone.0116820

**Published:** 2015-02-06

**Authors:** Harvey A. Whiteford, Alize J. Ferrari, Louisa Degenhardt, Valery Feigin, Theo Vos

**Affiliations:** 1 University of Queensland, School of Public Health, Herston, Queensland, Australia; 2 Queensland Centre for Mental Health Research, Wacol, Queensland, Australia; 3 University of Washington, Institute for Health Metrics and Evaluation, Seattle, Washington, United States of America; 4 UNSW Australia, National Drug and Alcohol Research Centre, New South Wales, Australia; 5 University of Melbourne, Melbourne School of Population and Global Health, Victoria, Australia; 6 Faculty of Health and Environmental Studies, National Institute for Stroke and Applied Neurosciences, AUT University, Auckland, New Zealand; “Mario Negri” Institute for Pharmacological Research, ITALY

## Abstract

**Background:**

The Global Burden of Disease Study 2010 (GBD 2010), estimated that a substantial proportion of the world’s disease burden came from mental, neurological and substance use disorders. In this paper, we used GBD 2010 data to investigate time, year, region and age specific trends in burden due to mental, neurological and substance use disorders.

**Method:**

For each disorder, prevalence data were assembled from systematic literature reviews. DisMod-MR, a Bayesian meta-regression tool, was used to model prevalence by country, region, age, sex and year. Prevalence data were combined with disability weights derived from survey data to estimate years lived with disability (YLDs). Years lost to premature mortality (YLLs) were estimated by multiplying deaths occurring as a result of a given disorder by the reference standard life expectancy at the age death occurred. Disability-adjusted life years (DALYs) were computed as the sum of YLDs and YLLs.

**Results:**

In 2010, mental, neurological and substance use disorders accounted for 10.4% of global DALYs, 2.3% of global YLLs and, 28.5% of global YLDs, making them the leading cause of YLDs. Mental disorders accounted for the largest proportion of DALYs (56.7%), followed by neurological disorders (28.6%) and substance use disorders (14.7%). DALYs peaked in early adulthood for mental and substance use disorders but were more consistent across age for neurological disorders. Females accounted for more DALYs in all mental and neurological disorders, except for mental disorders occurring in childhood, schizophrenia, substance use disorders, Parkinson’s disease and epilepsy where males accounted for more DALYs. Overall DALYs were highest in Eastern Europe/Central Asia and lowest in East Asia/the Pacific.

**Conclusion:**

Mental, neurological and substance use disorders contribute to a significant proportion of disease burden. Health systems can respond by implementing established, cost effective interventions, or by supporting the research necessary to develop better prevention and treatment options.

## Introduction

A substantial proportion of the world’s health problems in both high-income countries (HICs) and low-to-middle-income countries (LMICs) arises from mental, neurological, and substance use disorders [1,2]. Treatment rates for these disorders are low, particularly in LMICs, where treatment gaps of more than 90% have been documented. Even in HICs, where rates of treatment are comparatively higher, treatment for mental, neurological, and substance use disorders tends to be provided many years after the onset of the disorder [3,4].

Historically, major health policy decisions have been informed by mortality statistics. Although our understanding of diseases causing premature mortality expanded as a result, the lack of emphasis on morbidity undervalued the global impact of prevalent and disabling disorders with lower mortality, such as mental, neurological, and substance use disorders. Until recently, there was a poor understanding of the comparative global epidemiology of mental, neurological, and substance use disorders and slower progress compared to other disorders in identifying the most cost-effective interventions. Although these disorders exist in all countries, cultures also influence their development and presentation. The predominantly Western-based definitions of mental, neurological, and substance use disorders can be in conflict with cultural contexts[5], leading to challenges in assembling data on global epidemiology. For example, some languages do not have the words to describe concepts such as “sadness” or “depression” as they are described in Western countries. Epidemiological surveys in many LMICs tend to capture somatic manifestations of disorders such as depression and anxiety, which may not be as relevant to other countries and cultures [6–8]. Furthermore, explanations for the onset and progression of mental, neurological, and substance use disorders may be explained through mechanisms such the presence of spirits or curses rather than as medical disorders [5].

To improve the health outcomes of people with mental, neurological, and substance use disorders in both HICs and LMICs, it is important to understand not only the number and distribution of these disorders among countries, but also how they impact on health in terms of both mortality and disability, compared with other diseases and injuries. The first Global Burden of Disease Study (GBD 1990), which published data on disease burden in 1990 [9], reported that the category of mental, neurological, and substance use disorders—a grouping that included depression, selected anxiety disorders, bipolar disorder, schizophrenia, epilepsy, dementia, Parkinson’s disease, multiple sclerosis, alcohol, and drug use disorders—accounted for a significant proportion of the world’s disease burden, as measured by disability-adjusted life years (DALYs). The DALY is a health metric that captures the non-fatal component of the disease burden as years live with disability (YLDs), and the fatal component as years lost to premature mortality (YLLs) [9].

Findings from the Global Burden of Disease Study 2010 (GBD 2010), the most recent burden of disease study, were released in 2012. GBD 2010 was a comprehensive re-analysis of burden for 291 diseases and injuries and 67 risk factors [1,10–14]. It included the complete epidemiological reassessment of all diseases, injuries, and risk factors across 187 countries; 21 world regions; men and women; 1990, 2005, 2010; and 20 different age groups. Compared to GBD 1990, in GBD 2010 an expanded list of mental, neurological and substance use disorders were assessed. Rather than rely on a selective sample of data points, burden estimates were based on a systematic review of the literature to obtain all available epidemiological data. They were also derived using new statistical methods to model the epidemiological data, quantify disability, adjust for comorbidity between diseases, and propagate uncertainty [1,11]. Overall, GBD 2010 findings highlighted a shift in burden from communicable to noncommunicable diseases and from YLLs to YLDs [1,11]. Although communicable diseases remain a health priority in many LMICs, increasing life expectancies due to better reproductive health, childhood nutrition, and control of communicable diseases meant that more people in 2010 were living to ages where mental, neurological, and substance use disorders were most prevalent [15].

In GBD 2010, the burden of mental and substance use disorders were estimated separately from that of neurological disorders, such as dementia, Parkinson’s disease, and epilepsy. This approach was done to better accommodate differences in the burden between these groups of disorders. Mental and substance disorders were one of the leading causes of disease burden in 2010. They were responsible for 7.4% of global DALYs and 22.9% of global YLDs, making them the fifth leading cause of DALYs and the leading cause of YLDs [15]. Neurological disorders explained 3% of global DALYs and 5.6% of global YLDs [1,11]. The overarching findings of the study for all 291 diseases and injuries have been presented [1,10–14], as have findings for mental and substance use disorders [15,16]. This paper presents GBD 2010 burden estimates of mental, neurological and substance use disorders as a group. Analysing burden estimates at this aggregated level is important from both the clinical and population-health perspectives, given that the organization of services in many LMICs does not separate neurological disorders from mental disorders, something seen as a progression of Western medical subspecialization. Specifically, in this paper, we quantify the global disease burden attributable to mental, neurological, and substance use disorders and explore variations in burden by disorder type, age, gender, year, and region.

## Methods

This section summarizes how YLDs, YLLs, and DALYs were estimated for mental, neurological, and substance use disorders in GBD 2010. More detailed information about the input data and methods can be accessed elsewhere [8,15–23].

### Case Definition

Table 1 summarizes the mental, neurological, and substance use disorders in the GBD 2010 cause list. This included an extended list of disorders compared to previous GBD studies. To allow for comparability in measurement, the definitions of dementia, mental, and substance use disorders used for GBD 2010 were restricted to diagnostic classifications provided in the Diagnostic and Statistical Manual of Mental Disorders (DSM-IV-TR) [24] and the International Classification of Diseases (ICD-10) [25]. The epilepsy definition was based on ICD-10 [25].

**Table 1:** GBD 2010 Mental, Neurological, and Substance Use Disorders, Estimated Disability Weights, and Prevalent Cases.

Disorders	Disability weights	Prevalent cases (to the nearest 100,000)
Neurological disorders		
Alzheimer’s disease	mild: 0.082 (0.055–0.117); moderate:0.346 (0.233–0.475); severe:0.438 (0.299–0.584)	43,000,000
Parkinson’s disease	mild: 0.011 (0.005–0.021); moderate:0.263 (0.179–0.360); severe:0.549 (0.383–0.711)	5,100,000
Epilepsy	treated, seizure free:0.072 (0.047–0.106); treated, with recent seizures:0.319 (0.211–0.445); untreated:0.420 (0.279–0.572); severe:0.657 (0.464–0.827)	28,300,000
Multiple sclerosis	mild: 0.198 (0.137–0.278); moderate:0.445 (0.303–0.593); severe:0.707 (0.522–0.857)	1,800,000
Migraine	0.433 (0.287–0.593)	1,014,000,000
Tension-type headache	0.04 (0.025–0.062)	1,432,500,000
Substance use disorders		
Alcohol dependence	mild: 0.25(90.176–0.359); moderate:0.388(0.262–0.529); severe:0.549(0.384–0.708)	94,800,000
Opioid dependence	0.641 (0.459–0.803)	15,500,000
Cocaine dependence	0.376 (0.235–0.553)	6,900,000
Amphetamine dependence	0.353 (0.215–0.525)	17,200,000
Cannabis dependence	0.329 (0.223–0.455)	13,100,000
Mental disorders		
Major depressive disorder	mild: 0.159 (0.107–0.223); moderate:0.406 (0.276–0.551); severe:0.655 (0.469–0.816)	298,700,000
Dysthymia	0.159 (0.107–0.223)	105,700,000
Bipolar disorder	manic: 0.480 (0.323–0.642); depressive: 0.406 (0.276–0.551); residual: 0.035 (0.021–0.055)	58,900,000
Schizophrenia	acute: 0.756 (0.571–0.894); residual:0.576 (0.399–0.756)	21,500,000
Anxiety disorders	mild: 0.03 (0.017–0.048); moderate:0.149 (0.101–0.210); severe: 0.523 (0.365–0.684)	272,100,000
Eating disorders	Anorexia nervosa: 0.223 (0.151–0.313); Bulimia nervosa:0.223 (0.150–0.310)	Anorexia: 9,400,000 Bulimia: 8,600,000
Autism	0.259 (0.177–0.362)	14,900,000
Asperger’s syndrome	0.11 (0.073–0.157)	35,500,000
Attention-deficit hyperactivity disorder	0.049 (0.031–0.074)	36,400,000
Conduct disorder	0.236 (0.031–0.074)	48,700,000
Idiopathic intellectual disability	mild: 0.031 (0.018–0.049); moderate:0.08 (0.053–0.114); severe:0.126 (0.085–0.176); profound: 0.157 (0.107–0.221)	30,800,000

Source:[11,12,15,16]; Note: GBD 2010 comorbidity adjustments is applied to disability weights as opposed to prevalent cases so the latter cannot be summed across disorders that co-occur to avoid any double counting of cases; Prevalent cases were derived from estimates of point prevalence for all conditions apart from bipolar disorder and headaches where one-year prevalence was used. In addition to the disorders presented here, burden was estimated for three extra groupings of “other mental disorders”, “other drug use disorders,” and “other neurological disorders.” These were residual categories made up of rare disorders for which there were insufficient data to estimate prevalence. Instead, we estimated burden for these disorders using other available data for instance a ratio of YLDs to YLLs for similar or related disorders.

### Estimation of Years Lived with Disability

Unlike GBD 1990, which estimated incident-YLDs, GBD 2010 estimated prevalent-YLDs by multiplying the prevalence of a given condition by its disability weight, and without age-weighting and discounting, both of which had been used in earlier GBD studies[26]. As these, in combination with other factors, such as newly derived disability weights, changed the DALY metric from GBD 1990, the YLDs in GBD 2010 were recalculated for 1990, as well as for 2005 and 2010, to facilitate the investigation of changes in burden across time.

For each disorder, prevalence data were assembled by conducting systematic reviews of the published and gray literature to capture data on prevalence, incidence, remission, duration, and excess mortality [16,18,20,21,27–29]. DisMod-MR, a Bayesian meta-regression tool [11] developed specifically for GBD 2010, was then used to model prevalence by disorder type, age, gender, year, region and country. A generalized negative binomial model was estimated for all epidemiological data using super-region, region, and country random effects, as well as two sets of covariates: study level covariates that adjusted for systematic bias in the raw epidemiological data, and country level covariates that aided the predictive power of the model by adjusting for known ecological effects in the data, such as the effect of conflict or economic status on prevalence.

DisMod-MR also made use of the data available to estimate prevalence for countries and regions for which no raw data were available. Given that the aim of GBD 2010 was to compare burden caused by diseases and injuries between countries, this approach was considered preferable to the alternative, which was to entirely exclude parts of the world where no local data was available from GBD estimations, thereby assuming the prevalence of mental, neurological, and substance use disorders in those countries was zero. The final results provided prevalence estimates for 187 countries, 21 regions, 7 super regions, 20 age groups, men and women, for 1990, 2005, and 2010. Uncertainty around the raw epidemiological data was propagated to the final DisMod-MR model to provide 95% uncertainty intervals around all prevalence estimates [11].

Disability weights in GBD 2010 quantified the severity of any short- or long-term health loss. They ranged from zero to one, with zero equivalent to perfect health and one equivalent to death. Disability weights were estimated for 220 distinct health states that together represented the non-fatal consequences of diseases and injuries in the study. Population-based surveys in Bangladesh, Indonesia, Peru, Tanzania, and the United States, in addition to an open-internet survey (accessible in English, Mandarin, and Spanish), captured the views of 31,038 individuals. In each survey, participants were asked to compare two randomly selected health states and to identify which of the two they considered healthier. To calculate disability weights, their responses were anchored on a scale of zero to one, using a series of “population health equivalence” questions designed to compare overall health benefits of lifesaving or disease prevention programs[12]. For a number of mental, neurological, and substance use disorders, disability weights were generated for more than one health state to capture differences in disability within the symptomatic presentation of the disorder (see Table 1 for heath states investigated for each disorder). For major depressive disorder, for instance, disability weights were estimated for mild, moderate, and severe states. Survey data on the distribution of these health states in the population were then used to proportionally aggregate the three disability weights into an average disability weight for the disorder, which also took into consideration the proportion of those diagnosed with major depressive disorder who were asymptomatic at the time of survey [22]. For the headaches, data on the frequency and average duration of episodes were used to estimate a proportion of time symptomatic.

Finally, microsimulation methods were used to conduct a study-wide comorbidity correction for all GBD 2010 disability weights. For each age, gender, year, region and country category, a hypothetical population of 20,000 individuals was created who would have no, one, two, or more comorbid conditions, using prevalence data as probabilities. Using a multiplicative function, a combined disability weight was calculated for all comorbid health states and then reapportioned to each health state relative to the sum of comorbid disability weights. The average “corrected” disability for each health state was calculated in each age, gender, year, and country stratum and the decrement compared to the original disability weight taken as the comorbidity correction for YLDs [11].

### Estimation of Years of Life Lost to Premature Mortality

YLLs for each disorder were estimated by multiplying deaths occurring as a result of a given disorder, by the reference standard life expectancy at the age the death occurred. Standard life expectancy data came from GBD 2010 standard model life tables [12]. The number of deaths occurring as a result of each mental, neurological, and substance use disorder was estimated from cause of death data (by age, gender, year, region and country) available for 235 of 291 diseases and injuries in GBD 2010 [10]. These estimates were based on comprehensive searches of data sources such as vital registrations, verbal autopsies, and mortality surveillance databases, dating back to 1980, for 187 countries. Codes from different revisions of the ICD cause of death directory were matched to GBD 2010’s list of diseases and injuries. Deaths allocated to unclear or imprecise diagnoses (for example, deaths assigned to conditions that were not likely to be the underlying cause of death) were reassigned using standard algorithms [13]. Deaths and YLLs were estimated for schizophrenia, alcohol use disorders, drug use disorders, anorexia nervosa, epilepsy, Alzheimer’s disease, Parkinson’s disease, multiple sclerosis, and the residual groups of other mental, substance use, and neurological disorders. There were insufficient data for the remaining mental, neurological, and substance use disorders to enable the allocation of deaths to specific disorders.

### Estimation of Disability-Adjusted Life Years

For each disorder, YLDs and YLLs were summed to estimate DALYs. For disorders with insufficient data to estimate YLLs, YLDs were equated with DALYs. Uncertainty was estimated at all stages of the analysis through microsimulation methods and propagated to the final burden estimates. YLDs, YLLs, and DALYs in this paper are presented at the following levels:

- Global
- Disaggregated by disorder type, age, gender, and year.
- Disaggregated by GBD 2010’s seven super-region groups: East Asia and the Pacific, Eastern Europe and Central Asia, High-income regions (North America, Australasia, Western Europe, High-income Asia Pacific, and Southern Latin America), Latin America and the Caribbean, North Africa and the Middle East, South Asia, and Sub-Saharan Africa.
- Disaggregated by developed and developing regions.

The terms developed and developing regions are used here rather than high- and low-to-middle income regions for consistency with the presentation of GBD 2010 estimates. The classification of countries into regions and regions into super-regions was based on both geographical proximity and epidemiological likeness in terms of cause of death patterns [1,11]. Materials published by Whiteford and collaborators[15] provide a list of all countries in each region and super-region. Where age-standardized DALY rates are presented, these were estimated using direct standardization to the global standard population that the WHO proposed in 2001 (http://www.who.int/healthinfo/paper31.pdf).

## Results

**Fig 1:**
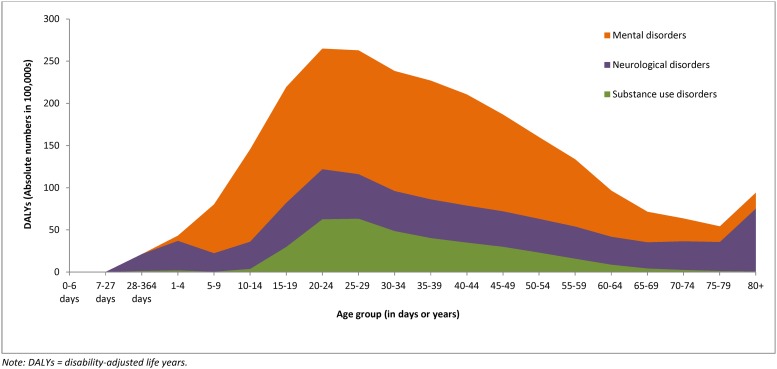
Absolute DALYs Attributable to Mental, Neurological, and Substance Use Disorders, by Age, 2010.

Mental, neurological, and substance use disorders accounted for 258 million DALYs in 2010, which was equivalent to 10.4% of total all-cause DALYs. Within mental, neurological, and substance use disorders, mental disorders accounted for the highest proportion of DALYs (56.7%), followed by neurological disorders (28.6%) and substance use disorders (14.7%). For all three groups of disorders, DALYs occurred across the lifespan (Fig. 1); however, there was a peak in early adulthood (between ages 20 and 30 years) for mental and substance use disorders compared to neurological disorders, where DALYs were more constant across age groups.

Absolute DALYs for mental, neurological, and substance use disorders increased by 41% between 1990 and 2010, from 182 million to 258 million DALYs. With the exception of substance use disorders, this increase was largely due to changes in population growth and aging, with DALY rates remaining fairly stable over time. Table 2 summarizes age-standardized DALY rates for 1990 and 2010.

**Table 2:** Age-Standardized DALY Rates Attributable to Mental, Neurological, and Substance Use Disorders, 1990 and 2010.

	Age standardized DALY rates (per 100,000)			
	Male		Female	
Disorder	1990	2010	1990	2010
**Neurological disorders**				
Alzheimer’s disease and other dementias	125.7	155.5	153.7	178.6
Parkinson’s disease	32.7	36.6	23.2	23.3
Epilepsy	261.6	269.3	226.0	232.9
Multiple sclerosis	16.3	12.3	23.7	19.8
Migraine	233.1	236.6	405.9	415.8
Tension-type headache	24.1	24.0	28.3	28.3
Other neurological disorders	228.0	259.9	200.0	266.7
**Substance use disorders**				
Alcohol use disorders	431.0	409.9	117.2	106.0
Opioid use disorders	139.0	184.4	63.8	78.4
Cocaine use disorders	22.5	22.0	10.3	9.7
Amphetamine use disorders	45.4	47.3	26.9	27.6
Cannabis use disorders	38.8	36.7	22.3	21.3
Other drug use disorders	83.7	97.0	44.6	47.9
**Mental disorders**				
Major depressive disorder	694.8	689.9	1171.7	1161.2
Dysthymia	135.3	135.8	189.7	190.0
Bipolar affective disorder	172.0	172.1	204.6	204.8
Schizophrenia	230.7	223.0	187.8	180.6
Anxiety disorders	274.3	273.0	508.9	510.3
Eating disorders	4.4	3.9	47.6	59.5
Autism	85.1	85.8	29.5	29.6
Asperger’s syndrome	85.2	85.0	20.3	20.3
Attention-deficit hyperactivity disorder	10.8	10.6	3.1	3.1
Conduct disorder	111.9	113.3	47.0	47.6
Idiopathic intellectual disability	25.3	17.7	18.2	11.9
Other mental and behavioral disorders	25.5	23.3	21.5	20.8

Table 3 summarizes DALYs assigned to each mental, neurological, and substance use disorder in 2010. These disorders as a group ranked as the third leading cause of DALYs after cardiovascular and circulatory diseases (explaining 11.9% of DALYs) and diarrhoea, lower respiratory infections, meningitis, and other common infectious diseases (explaining 11.4% of DALYs). The greatest variation in burden within the disorder groupings was for mental disorders. Major depressive disorder was responsible for the highest proportion of mental, neurological, and substance use disorder DALYs (24.5%); attention-deficit hyperactivity disorder was responsible for the lowest (0.2%).

**Table 3:** DALYs (absolute numbers, and proportions) attributable to mental, neurological and substance use disorders in 2010.

Disorder	Absolute DALYs (to nearest 100,000)	Proportion of total (all cause) DALYs (%)	Proportion of mental, neurological, and substance use DALYs (%)
**Neurological disorders**			
Alzheimer’s disease and other dementias	11,400,000	0.5	4.4
Parkinson’s disease	1,900,000	0.1	0.7
Epilepsy	17,400,000	0.7	6.8
Multiple sclerosis	1,100,000	0.04	0.4
Migraine	22,400,000	0.9	8.7
Tension-type headache	1,800,000	0.1	0.7
Other neurological disorders	17,900,000	0.7	6.9
**Substance use disorders**			
Alcohol dependence	17,700,000	0.7	6.9
Opioid dependence	9,200,000	0.4	3.6
Cocaine dependence	1,100,000	0.04	0.4
Amphetamine dependence	2,600,000	0.1	1.0
Cannabis dependence	2,100,000	0.1	0.8
Other drug use disorders	5,100,000	0.2	2.0
**Mental disorders**			
Major depressive disorder	63,200,000	2.5	24.5
Dysthymia	11,100,000	0.4	4.3
Bipolar disorder	12,900,000	0.5	5.0
Schizophrenia	13,600,000	0.5	5.3
Anxiety disorders	26,800,000	1.1	10.4
Eating disorders	2,200,000	0.1	0.9
Autism	4,000,000	0.2	1.6
Asperger’s syndrome	3,700,000	0.1	1.4
Attention-deficit hyperactivity disorder	500,000	0.02	0.2
Conduct disorder	5,800,000	0.2	2.2
Idiopathic intellectual disability	1,000,000	0.04	0.4
Other mental disorders	1,500,000	0.1	0.6

Note: DALYs presented have been aggregated across all countries, gender, and age groups for 2010.

**Fig 2:**
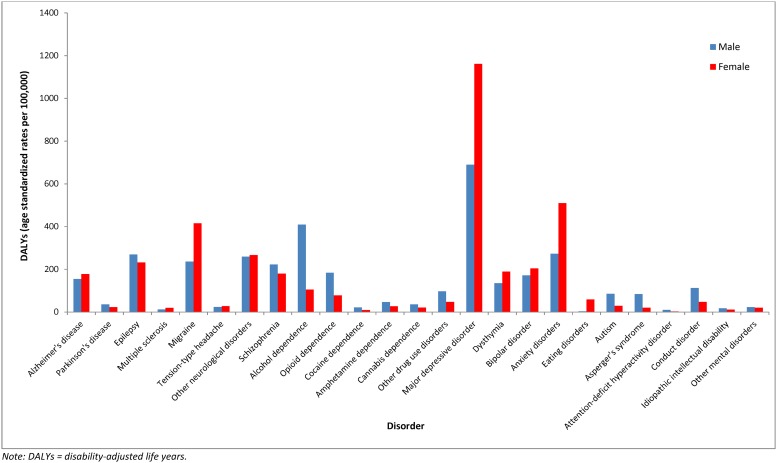
Age-Standardized DALY Rates Attributable to Individual Mental, Neurological, and Substance Use Disorders, by Gender, 2010.

Overall, in 2010, 124 million mental, neurological, and substance use DALYs occurred among males and 134 million among females. Fig. 2 shows DALY rates for each mental, neurological, and substance use disorder by gender. Women accounted for more DALYs in most of the mental and neurological disorders, except for mental disorders occurring in childhood, schizophrenia, Parkinson’s disease, and epilepsy, where men accounted for more DALYs. Men also accounted for more DALYs than women in all substance use disorders.

**Fig 3:**
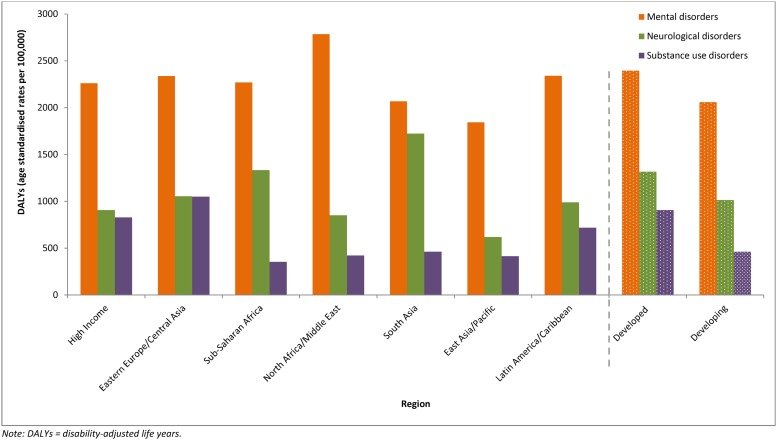
Age-Standardized DALY Rates Attributable to Mental, Neurological, and Substance Use Disorders, by Region, 2010.

Fig. 3 shows the burden attributable to mental, neurological, and substance use disorders in 2010 by GBD 2010’s super-region groupings, and by developed and developing world regions. Overall, the burden of these disorders was approximately 1.3 times higher in developed regions (15.5% of total DALYs) compared to developing regions (9.4% of total DALYs). When disaggregated by GBD super-regions, the burden (age standardized rate) of mental, neurological, and substance use disorders was highest in Eastern Europe and Central Asia and lowest in East Asia and Pacific. Mental disorders maintained the highest proportion of DALYs in all super-regions. The greatest variation in DALYs occurred within substance use disorders, where DALYs were almost three times higher in Eastern Europe and Central Asia, compared to Sub-Saharan Africa, where DALYs were lowest.

**Fig 4:**
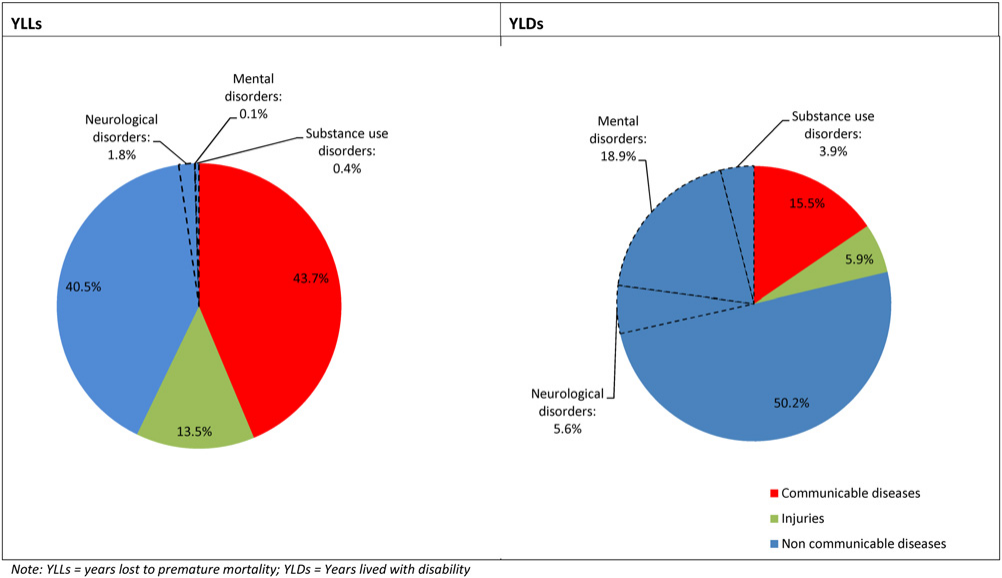
Proportion of Global YLDs and YLLs Attributable to Mental, Neurological, and Substance Use Disorders, 2010.

Fig. 4 illustrates the decomposition of global burden by YLDs and YLLs for communicable diseases, noncommunicable diseases, and injuries. Noncommunicable diseases explained a large proportion of YLDs and YLLs in 2010, when compared to communicable diseases and injuries. Within this group, mental, neurological, and substance use disorders were responsible for 28.5% of all YLDs, making them the leading cause of YLDs worldwide. In comparison, they contributed to only 2.3% of YLLs. Deaths and YLLs could be assigned to a mental, neurological, and substance use disorder only when the disorder was considered as a direct cause of death in the ICD cause of death directory. Using this approach, the majority of excess deaths in individuals with a mental disorder, in particular, were coded to the direct physical cause of death (for example, suicide deaths were coded under injuries as self-harm) rather than to the disorder.

## Discussion

Mental, neurological, and substance use disorders are a leading cause of the disease burden worldwide, substantially contributing to health loss across the lifespan. DALY rates are lower in developing compared to developed regions. However, population growth and a changing age profile are producing a shift in the disease burden from communicable to noncommunicable diseases and from YLLs to YLDs. This demographic and epidemiological transition is contributing to a rise in the burden of mental, neurological, and substance use disorders, particularly in developing regions.

Geographic variations exist within DALY rates. Mental disorder DALYs are highest in North Africa and the Middle East, substance use disorder DALYs are highest in Eastern Europe and Central Asia, and neurological disorders DALYs are highest in Asia South. These regional differences were driven by the global distribution of disorder prevalence and, in some instances, deaths. Analysis of GBD 2010 prevalence data for mental disorders, for example, highlighted the effect of conflict status on estimates. The prevalence of major depressive disorder and anxiety disorders was highest in countries with a history of conflict or war, many of which are in North Africa and Middle East [17,22]. The prevalence of opioid and cannabis dependence was highest in Australasia and Western Europe [19,20]. Cocaine dependence was highest in North America, High-income, and Southern Latin America. Although there was less regional variation in the prevalence of amphetamine dependence, the rates were highest in South East Asia and Australasia [17].

The largest contributor of YLLs for substance use disorder was opioid dependence, with particularly high proportions of deaths due to opioid dependence occurring in the North America, High-income, Europe Eastern, and Sub-Saharan Africa Southern. In many Eastern European and Sub-Saharan African countries, access to interventions found to be effective in reducing the risk of mortality from opioid dependence—such as opioid substitution therapy, needle and syringe programs, and HIV treatment for those who are HIV-positive—is limited. Access to these interventions in North America, High-income varies subnationally, with insufficient data to determine the access rates at the national level [19]. Prevalence and deaths attributable to Alzheimer’s disease were highest in North America, High-income, Europe Western, and Australasia. In contrast, prevalence and deaths attributable to epilepsy was highest in Sub-Saharan Africa.

Mental, neurological and substance use disorders rarely occur in isolation and can increase one’s risk of other diseases and injuries. This has significant consequences on life expectancy [30,31]. Previous studies have shown that up to 80% of deaths in those with mental, neurological and substance use disorders occur as a result of a comorbid physical illness such as cardiovascular disease or cancer [30,31]. For disorders like epilepsy, rates of excess-mortality also vary by world regions with higher rates reported in some developing countries [32]. In spite of this, YLDs explained a larger proportion of the burden due to mental, neurological and substance use disorders compared to YLLs in GBD 2010. To estimate YLLs, GBD 2010 followed the ICD-10 cause of death categories, whereby deaths can only be assigned to a given condition when the disorder is considered a direct cause of death. This approach can only account for some of the excess deaths attributable to mental, neurological, and substance use disorders, given that deaths will also be coded to the direct physical cause of death. For instance, ischemic heart disease or suicide deaths occurring as a result of mental, neurological and substance use disorders will be coded to cardiovascular disease and injuries rather than to the mental, neurological or substance use disorder.

The additional burden attributable to mental, neurological, and substance use disorders as a risk factor for other health outcomes can be investigated through comparative risk assessment analysis, which compares the current health status to a theoretical-minimum-risk exposure, in this case, the counterfactual status of the absence of mental, neurological, and substance use disorders in the population. The use of this method to estimate the additional burden due to mental and substance use disorders as risk factors for suicide showed that these disorders could account for over 50% of suicide YLLs in GBD 2010. These additional suicide YLLs would have increased the overall burden of mental and substance use disorders in 2010 from 7.4% to 8.3% of global DALYs [33].

Although not adopted in GBD 2010, the use of age weighting in many economic analyses and in earlier GBD studies[34] recognizes and attempts to incorporate the social preference for avoiding health loss in young adults. In spite of its absence in GBD 2010 estimates, the peak impact of mental, neurological, and substance use disorders in early adulthood remained and demonstrated the ubiquitous effect of these disorders at a time of life when individuals are starting to make significant social and economic contributions to their families and societies. Although the peak burden of mental, neurological, and substance use disorders is found in young adults, there is, unlike many chronic diseases, a significant burden in children and younger adolescents. For countries such as those in Sub-Saharan Africa where children constitute 40% of the population [35], these findings highlight the need for prevention and treatment services targeted to children and adolescents. The availability of such services is often more sporadic than for adult services.

Within the mental, neurological, and substance use disorder group, particular disorders make a disproportionate contribution to the burden; for these, the need for cost-effective interventions is highest. Depressive and anxiety disorders are very prevalent and are significant contributors to the burden. Treatment rates for these disorders are low in most countries, as they are for epilepsy, migraine, alcohol, and opioid dependence, the other large contributors to disease burden.

Where cost-effective prevention or treatment of mental, neurological, and substance use disorders is less attainable, services can still provide treatment and support that mitigate their impacts. Pharmacological and psychosocial interventions may assist individuals with schizophrenia to live a better quality of life. Treatment and support services may alleviate the impact of dementia, a disorder that has to be addressed most urgently in countries with rapidly aging populations.

Although it represents the most comprehensive assessment of the burden due to mental, neurological, and substance use disorders to date, not all elements of the burden were captured. By focusing on health loss, burden in GBD 2010 does not extend to welfare loss; hence, it does not capture all of the consequences of mental, neurological, and substance use disorders for families or societies. Disability weights in GBD 2010 were derived from surveying the general population (rather than by clinicians, as in previous GBD studies), with the aim of better capturing the societal view of health loss. Nevertheless, adequately encompassing the complexity of health states that represent mental, neurological, and substance use disorders within the survey was challenging; the extent to which the GBD 2010 disability weights entirely reflected the associated health loss is an important area for further research. Finally, the established definitions of mental, neurological, and substance use disorders used in the study may not be sensitive to non-Western presentation of these disorders, which may have led to an underestimation of burden in developing regions. A task for upcoming GBD analyses will be to explore the extent to which certain disorders are misdiagnosed as other mental or physical disorders in developing countries and the consequence on burden.

## Conclusions

Mental, neurological, and substance use disorders contribute to a significant proportion of the global burden of disease and will continue to do so as the shift in burden from communicable to noncommunicable diseases continues. Health systems worldwide need to respond to this rising burden by implementing proven, cost-effective interventions; where these are limited, it will be important to support the research necessary to develop better prevention and treatment options.
